# Individual or combined transcatheter arterial chemoembolization and radiofrequency ablation for hepatocellular carcinoma: a time-to-event meta-analysis

**DOI:** 10.1186/s12957-021-02188-4

**Published:** 2021-03-19

**Authors:** Chuang Jiang, Gong Cheng, Mingheng Liao, Jiwei Huang

**Affiliations:** 1 Department of Liver Surgery, Liver Transplantation Center, West China Hospital, Sichuan University, Chengdu, Sichuan China; 2 Department of Gastroenterology, Cadre Ward, Gansu Provincial Hospital, lanzhou, Gansu China

**Keywords:** Hepatocellular carcinoma, Transcatheter arterial chemoembolization, Radiofrequency ablation, Combined treatment, Meta-analysis

## Abstract

**Background:**

There is still some debate as to whether transcatheter arterial chemoembolization (TACE) plus radiofrequency ablation (RFA) is better than TACE or RFA alone. This meta-analysis aimed to compare the efficacy and safety of TACE plus RFA for hepatocellular carcinoma (HCC) with RFA or TACE alone.

**Methods:**

We searched PubMed, MEDLINE, Embase, Cochrane Library, and CNKI (China National Knowledge Infrastructure) for all relevant randomized controlled trials and retrospective studies reporting overall survival (OS), recurrence-free survival (RFS), and complications of TACE plus RFA for HCC, compared with RFA or TACE alone.

**Results:**

Twenty-one studies involving 3413 patients were included. TACE combined with RFA was associated with better OS (hazard ratio [HR]=0.62, 95% confidence intervals [CI] = 0.55–0.71, *P* < 0.001) and RFS (HR = 0.52, 95% CI = 0.39–0.69, *P* < 0.001) than TACE alone; compared with RFA alone, TACE plus RFA resulted in longer OS (HR = 0.63, 95% CI = 0.53–0.75, *P* < 0.001) and RFS (HR = 0.60, 95% CI = 0.51–0.71, *P* < 0.001). Subgroup analyses by tumor size also showed that combined treatment resulted in better OS and RFS compared with RFA alone in patients with HCC larger than 3 cm. Combined treatment resulted in similar rate of major complications compared with TACE or RFA alone (OR = 1.78, 95% CI = 0.99–3.20, *P* = 0.05; OR = 1.00, 95% CI = 0.42–2.38, *P* = 1.00, respectively).

**Conclusions:**

TACE combined with RFA was more effective for HCC than TACE alone. For patients with a tumor larger than 3 cm, the combined treatment also achieved a better effect than RFA alone.

**Supplementary Information:**

The online version contains supplementary material available at 10.1186/s12957-021-02188-4.

## Background

Hepatocellular carcinoma (HCC) is the most common primary liver cancer [1], is the fourth leading cause of cancer death in the world, and ranks the fifth among all the diseases in the world [2]. Hepatocellular carcinoma often occurs in patients with a background of cirrhosis and is an important cause of death in patients with cirrhosis, and the common causes of cirrhosis vary from country to country, such as excessive alcohol intake [3], schistosomiasis infection, and nonalcoholic fatty liver disease (NAFLD) in developed regions [4], while in China, the main cause is hepatitis B infection (HBsAg+). There are multiple staging systems to assess HCC prognosis and guide treatment [5], such as Tumour, Node, Metastasis (TNM); The Cancer of the Liver Italian Program; the Hong Kong Liver Cancer staging system [6]; and the Barcelona Clinic Liver Cancer (BCLC) staging system, which is the most widely used staging system for hepatocellular carcinoma. Patients with hepatocellular carcinoma at each stage have one or more treatment options that are relatively most suitable. But with the diversification of treatment options, including surgical resection, radiofrequency ablation (RFA), transcatheter arterial chemoembolization (TACE), microwave ablation, targeted therapy, and transplantation, it is worth studying by comparing which one is the best, so as to identify candidates who are suitable for a certain treatment [1]. Surgical resection has been recommended for most HCC patients [7]. However, due to the absence of specific symptoms in the initial stage of HCC, early diagnosis is difficult, so less than 20% of patients have surgical indications at the time of diagnosis. While for early-stage HCC, the radiofrequency ablation (RFA) is micro-invasive and with less pain, shorter length of hospital stay at a lower cost [8], and similar benefits to surgical resection, but there are always concerns of tumor residual which might lead to early tumor recurrence [9–12]. The effect of RFA is affected by tumor size and the “heat-sink” effect [13].

TACE has been applied in HCC patients for more than 40 years [14]. Though HCC is not sensitive to most systemic chemotherapy, adjuvant TACE after localized HCC treatment is related to better patient survival outcome according to previous researches [15–18] and meta-analysis [19]. As TACE could trigger tumor necrosis after tumor hypoxia, adjuvant TACE combined with RFA may theoretically bring about better outcomes. A number of published meta-analyses had compared adjuvant RFA with large HCC tumor after TACE therapy [20–22], but their results could hardly been utilized in directing clinical treatment because the complicated factors related to succeeding down-stage treatment could not be homogenized. Several years ago, Liu et al. published a meta-analysis of adjuvant TACE treatment after RFA [23], only seven studies were included. Many high-quality studies have been published in recent years, providing more information for detailed statistical analysis. Thus, this up-to-date meta-analysis aimed to evaluate the effectiveness of adjuvant TACE therapy combined with RFA in improving the outcome of patients with primary HCC.

## Methods

### Search strategy

As of June 31, 2020, studies were identified through a search of PubMed, MEDLINE, Embase, Cochrane Library, and CNKI. The retrieval strategy included subject words and free words. We combined the terms such as “HCC,” “hepatocellular carcinoma,” or “liver cancer”; “radiofrequency ablation” or “RFA”; and “Therapeutic Chemoembolization,” “transarterial chemoembolization,” “transcatheter arterial chemoembolization,” or “TACE.” No language limitations were imposed in our search.

### Inclusion and exclusion criteria

Studies were included in the analysis if (1) either randomized controlled trials (RCTs) or observational studies comparing combination therapy of TACE and RFA versus RFA or TACE alone for HCC; (2) HCC can be diagnosed with CT or MRI if the typical characteristics are present; (3) full-text, or abstract and figures available; (4) providing outcome as OS or RFS, with comparisons between the outcomes of TACE and RFA with TACE or RFA alone; and (5) patients received comparable treatments except for TACE or RFA in specific settings. If studies were duplicates, the one with complete data was included.

Studies were excluded if they were published only in the form of case reports, editorials, reviews, and conference abstracts.

### Quality assessment

The Cochrane Collaboration’s tool was used to assess the risk of bias among RCTs, considering random sequence generation, allocation concealment, blinding of participants and personnel, blinding of outcome assessment, incomplete outcome data, and selective reporting [24]. Observational studies were assessed by the Newcastle-Ottawa Quality Assessment Scale (NOS) [25]. This score assesses studies according to the selection of patients in the exposed and the non-exposed group, comparability of the two groups, and outcome of the single studies. A study can be rated 0–9 stars based on these criteria while 6 stars or above was considered high quality in previous studies and was included in this review.

Publication bias was evaluated by funnel plots, Begg’s test, and Egger’s test [26]. The funnel plot is a widely used tool within meta-analysis for detecting publication bias [27]. It has the advantage of visually presenting the results through graphics, and the obvious asymmetry of funnel plot indicates a large publication bias in meta-analysis [28]; in the absence of bias, it should be a symmetrical funnel plot [29] but it cannot be quantitatively detected, and Begg’s test and Egger’s test can conduct a quantitative test of publication bias. When there were fewer included studies, Egger’s test was more effective than Begg’s test [30]. In order to make the detection of publication bias more comprehensive, we used the above three methods together to make a comprehensive assessment.

### Statistical extraction and analysis

The hazard ratio (HR) and its 95% confidence interval (CI) were used as an indicator of time-to-event to assess the pooled effects. HR and 95% CI were extracted from the included studies. If both single factor and multivariate analysis data were available in the original research, the results of the multivariate analysis were selected to reduce the interference of confounding factors. If an article did not provide HR and 95% CI for OS or RFS, we extracted the HR from Kaplan-Meier curves according to the method described by Tierney [31]. In addition, complications were compared by calculating odds ratio (OR) with 95% CI. We used Cochrane’s *Q* statistic to assess heterogeneity between studies [32]. The *Q*-test and *I*^2^ were utilized to define the heterogeneity, according to the Cochrane Handbook. The value of *I*^2^ranges from 0 to 100%, and the heterogeneity increases with the increase of value, a value of *I*^2^of 0–25% indicates insignificant heterogeneity, 25–50% indicates low heterogeneity, 50–75% indicates moderate heterogeneity, and > 75% indicates high heterogeneity [33, 34]. In cases of *P* ≥ 0.1 or *I*^2^ < 50%, indicating that heterogeneity was within the acceptable range, the fixed effects model was used to pool the results. Because the application condition of fixed effects model is more severe than that of random effects model. Only when the heterogeneity is within an acceptable range, the results obtained by using fixed effects model can be reliable; otherwise, the random effects model was used. In addition, funnel plots, Begg’s test, and Egger’s test were used to evaluate publication bias, and sensitivity analysis was used to test the stability of the pooled effects [35]. For all analyses, *P* < 0.05 was considered statistically significant. RevMan 5.2 (Copenhagen: The Nordic Cochrane Centre, The Cochrane Collaboration, 2014) and Stata 12 (StataCorp LP, Stata Statistical Software, College Station, TX, USA) were used for the statistical analysis (Supplementary Figure 1).

## Results

### Study selection and quality evaluation

**Fig. 1:**
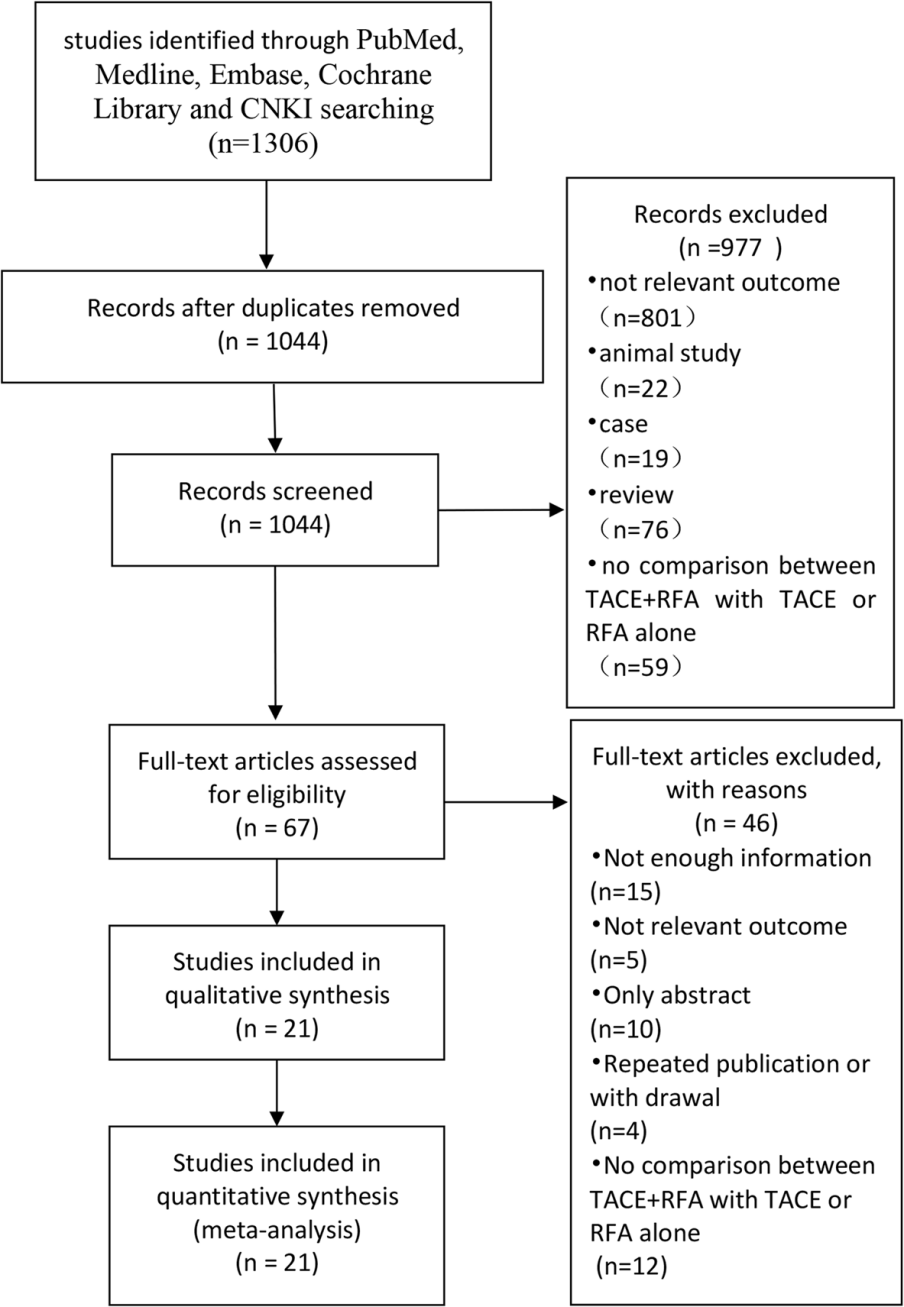
The flow chart represents the screening process

There were 1306 studies identified after searching PubMed, MEDLINE, Embase, Cochrane Library, and CNKI. Following the initial review, 262 repetitive documents were eliminated by automatic deduplication and manual removal. Reading of the title and abstract led to the exclusion of 977 studies. After the full text was read, 21 articles were finally included in the study based on reasonable criteria (Fig. 1). There were 10 studies comparing TACE+RFA and RFA alone for HCC, and 15 studies comparing TACE+RFA and TACE alone. Table 1 shows the basic characteristics of the 21 studies. As detailed in the Cochrane Handbook, three RCTs were evaluated with the Cochrane Collaboration’s tool (Supplementary Figure 2). Because of problems such as treatment and ethics, double blindness is difficult to achieve in studies of this type. The 18 cohort studies were assessed with the NOS (Supplementary Table 1). The scores of these studies are all greater than or equal to 7 points.

**Table 1:** The basic characteristics included in this meta-analysis

Study	Country	Type	Study arms	NP	Age	Gender (M/F)	Tumor size (cm)	Child-Pugh(A/B/C)	HBsAg+	HCV-Ab+	NOS score
Shibata et al. [15]	Japan	Cohort study	TACE+RFA	46	67.2±8.9	31/15	1.7±0.6 (0.9–3.0)	32/14/0	12	32	********
			RFA	43	69.8±8.0	33/10	1.6±0.5 (0.8–2.6)	33/10/0	9	30	
Yang et al. [36]	China	Cohort study	TACE+RFA	31	57.8 (43.0–78.0)	24/7	3.5	20/10/1	NA	NA	********
			TACE	35	51.2 (30.0–74.0)	30/5	3.6	21/13/1	NA	NA	
			RFA	37	58.3 (38.0–80.0)	27/10	3.8	23/13/1	NA	NA	
Morimoto et al. [37]	Japan	RCT	TACE+RFA	19	70.0 (57.0–78.0)	15/4	3.6±0.7	18/1/0	0	17	-
			RFA	18	73.0 (48.0–84.0)	12/6	3.7±0.6	16/2/0	0	16	
Kim et al. [16]	South Korea	Cohort study	TACE+RFA	83	59.7±10.4	69/14	2.5±0.3	67/16/0	50	11	********
			RFA	231	58.0±10.1	182/49	2.4±0.3	170/61/0	158	19	
Peng et al. [38]	China	RCT	TACE+RFA	69	57.5±10.0 (19.0–75.0)	59/9	NA	60/9/0	63	NA	-
			RFA	70	55.1±9.5 (22.0–75.0)	55/15	NA	59/11/0	65	NA	
Lin et al. [39]	China	Cohort study	TACE+RFA	32	64.9±8.8	24/8	4.09±0.55	NA	NA	NA	*******
			RFA	30	60.1±10.2	23/7	3.94±0.54	NA	NA	NA	
Peng et al. [40]	China	RCT	TACE+RFA	94	53.3±11	75/19	3.47±1.44	90/4/0	85	6	-
			RFA	95	55.3±13.3	71/24	3.39±1.35	90/5/0	83	6	
Liu et al. [41]	China	Cohort study	TACE+RFA	45	45.0–75.0	36/9	4.0–15.0	13/20/12	NA	NA	*******
			TACE	43	44.0–78.0	34/9	5.0–14.0	10/23/10	NA	NA	
Yin et al. [42]	China	Cohort study	TACE+RFA	55	NA	47/8	5.9 (5.0–8.0)	48/7/0	36	NA	********
			TACE	156	NA	138/18	6.0 (5.0–8.0)	136/20/0	118	NA	
Gao et al. [43]	China	Cohort study	TACE+RFA	35	48.6±10.4	31/4	6.48±1.25	24/11/0	27	NA	********
			TACE	32	51.8±11.0	29/3	7.05±1.47	25/7/0	26	NA	
Hyun et al. [44]	Korea	Cohort study	TACE+RFA	37	57.7±7.7	31/6	NA	34/3/0	30	1	********
			TACE	54	59.5±9.5	42/12	NA	45/9/0	45	6	
Shi et al. [45]	China	Cohort study	TACE+RFA	31	64.0 (39.0–48.0)	24/7	NA	29/2/0	NA	NA	********
			TACE	43	64.0 (39.0–48.0)	34/9	NA	39/4/0	NA	NA	
Song et al. [46]	Korea	Cohort study	TACE+RFA	87	60.4 (29.1–78.0)	70/17	2.5 (1.0–4.6)	80/7/0	58	21	********
			TACE	71	60.0 (23.0–87.2)	53/18	2.5 (1.0–4.7)	68/3/0	49	14	
			RFA	43	62.0 (35.0–88.0)	31/12	2.2 (1.3–4.7)	37/6/0	28	9	
Tang et al. [47]	China	Cohort study	TACE+RFA	40	48.28±13.48	29/11	5.35±1.10	18/22/0	16	9	********
			TACE	43	45.84±15.08	33/10	5.64±1.41	19/24/0	23	7	
			RFA	49	47.14±13.27	34/15	5.78±1.35	22/27/0	26	12	
Kim et al. [48]	Korea	Cohort study	TACE+RFA	105	63.4±9.7	82/23	2.83±0.76	98/7/0	71	17	*********
			TACE	102	62.4±10.2	81/21	2.87±0.92	82/20/0	60	20	
Zhu et al. [49]	China	Cohort study	TACE+RFA	35	47.5±10.3	26/9	5.97±1.28	24/11/0	NA	NA	********
			TACE	37	48.1±10.8	29/8	6.02±1.31	28/9/0	NA	NA	
Shimose et al. [50]	Japan	Cohort study	TACE+RFA	68	70.5 (46–89)	26/42	3.27 (2.1–5.8)	NA	4	57	********
			TACE	68	71 (48–85)	27/41	3.14 (1.0–8.5)	NA	10	50	
Liu et al. [51]	China	Cohort study	TACE+RFA	209	59.2 ± 4.0 (18–75)	184/25	NA	189/20/0	180	10	********
			TACE	195	58.7 ± 4.0 (20–75)	165/30	NA	180/15/0	176	7	
Lee et al. [52]	Korea	Cohort study	TACE+RFA	82	60.3 ± 10.6	60/22	1.77±0.60	77/5/0	58	15	********
			TACE	85	60.6 ± 10.3	59/26	1.91±0.62	76/9/0	64	5	
Chu et al. [53]	Korea	Cohort study	TACE+RFA	109	58.4 ± 10.2	83/26	3.7±0.5	93/16/0	79	17	********
			TACE	314	60.5 ± 10.6	224/90	3.8±0.5	254/60/0	221	40	
			RFA	115	61.1 ± 10.8	90/25	3.5±0.4	83/32/0	74	17	
Endo et al. [54]	Japan	Cohort study	TACE+RFA	46	74.0 (46.0–87.0)	35/11	3.2 (1.2–4.8)	36/10/0	5	27	********
			TACE	46	74.0 (54.0–89.0)	30/16	3.4 (1.1–4.9)	31/15/0	3	24	

*TACE* transcatheter arterial chemoembolization, *RFA* radiofrequency ablation, *NP* number of patients, *NA* not applicable, *M/F* male/famale, *RCT* randomized control trial, *NOS* Newcastle-Ottawa scale

Tabel 2 Subgroup analysis based on the tumor size and the age

### Descriptive statistics analysis of clinicopathological characteristics

Then, a descriptive statistics analysis of clinicopathological characteristics of patients in 21 included studies was performed (Supplementary Table 2). A total of 2339 patients were included in the TACE+RFA vsTACE group, while 1341 patients were included in the TACE+RFA vs RFA group. Because the indicators reported by various institutes are not exactly the same, when we make statistical analysis of an indicator, only the studies with reported related indicators were analyzed. For the TACE+RFA vs TACE group, there were no statistical differences in age (*p* = 0.919), gender (*p* = 0.401), HBsAg+ (*p* = 0.097), and Child-Pugh classification (*p* = 0.188) between the groups. However, the proportion of countries in TACE+RFA group was Japan, Korea, and China which were 11.2%, 41.4%, and 47.4%, respectively, compared with TACE group’s 8.6%, 47.3%, and 44.1%, respectively (*p*=0.007); tumor size (3.23 ± 1.24 vs 3.07 ± 1.26, *p* = 0.03); HCV-Ab+ (*p* = 0.019). However, TACE+RFA vs RFA group also had a significant difference in proportion of countries (10.7%, 45.7%, 43.6% vs 8.3%, 53.2%, 38.5%, *p* = 0.02), tumor size (3.66 ± 1.64 vs 4.03±1.66, *p* < 0.001), HCV-Ab+ (*p* = 0.04), and Child-Pugh classification (*p* = 0.013).

### Overall survival

**Fig. 2:**
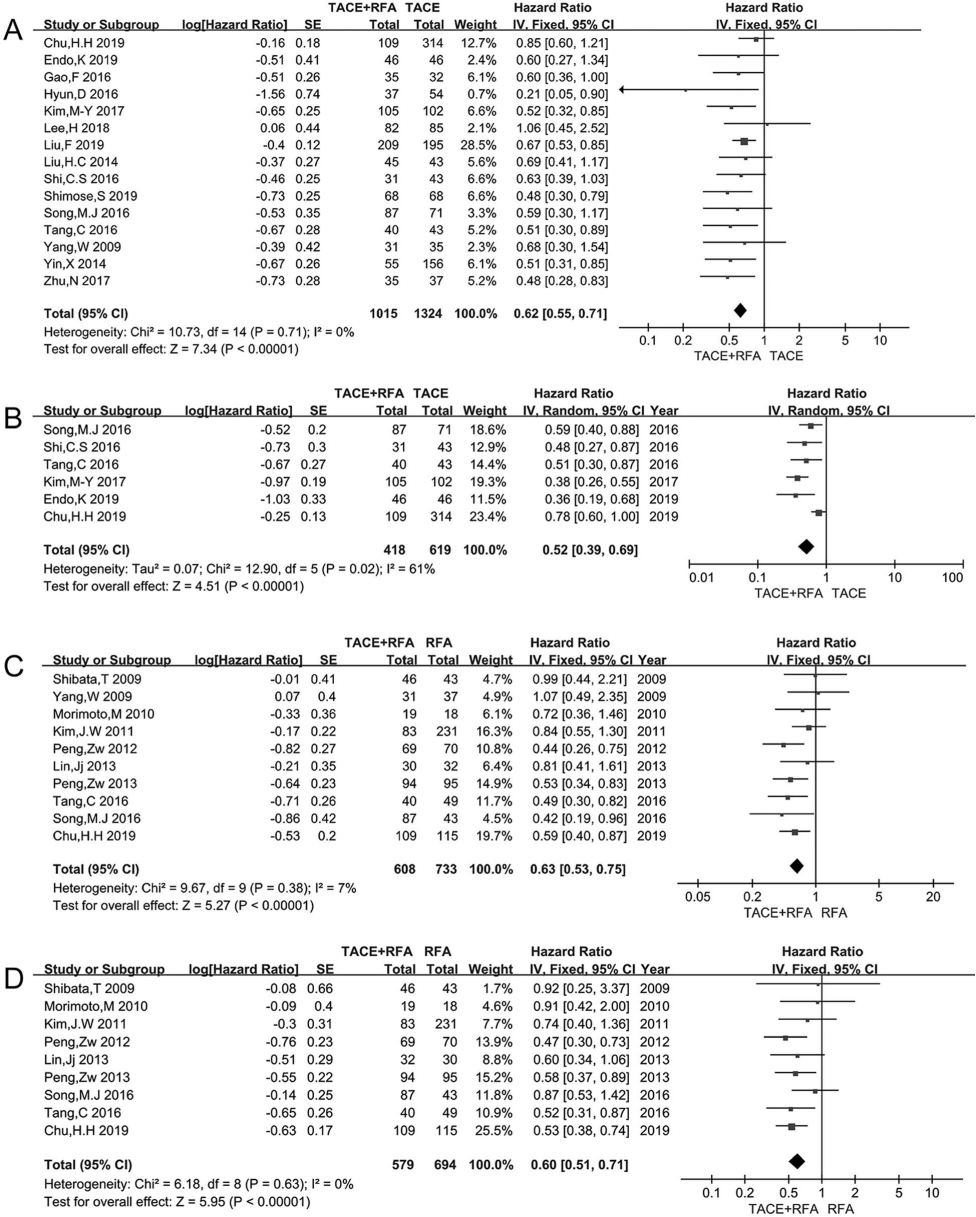
**a** OS of TACE+RFA vs TACE; **b** RFS of TACE+RFA vs TACE; **c** OS of TACE+RFA vs RFA; **d** RFS of TACE+RFA vs RFA

Ten studies including a total of 2339 patients compared the OS of TACE+RFA and TACE alone. The heterogeneity test showed *p* = 0.71, *I*^2^ = 0%. Therefore, the fixed effects model was selected to pool the HR of OS. The pooled result showed that the OS of TACE combined with RFA was better than that of TACE alone (HR = 0.62, 95% CI 0.55–0.71, *p* < 0.001). TACE+RFA was associated with a 38% lower hazard of death than that of TACE alone (Fig. 2a) (Supplementary Table 3).

The OS of TACE+RFA was compared with that of RFA in eight studies that included a total of 1341 patients. The heterogeneity test showed *p*=0.38, *I*^2^=7%. The fixed effects model was used to pool the results. The difference in OS was statistically significant (HR = 0.63, 95% CI 0.53–0.75, *p* < 0.001) (Fig. 2c) (Supplementary Table 3).

### Recurrence-free survival

Only six studies, including 1037 patients, compared the RFS of TACE+RFA and TACE alone. The heterogeneity test showed *p* = 0.02, *I*^2^ = 61%. So, the random effects model was used to pool the results. The difference in the RFS was statistically significant (HR = 0.52, 95% CI = 0.39–0.69, *p* < 0.001) (Fig. 2b) (Supplementary Table 3).

Nine studies with 1273 patients reported the RFS for TACE+RFA vs RFA alone (*p* = 0.63, *I*^2^ = 0%). Pooled results showed that the combined treatment group achieved better RFS than the RFA alone group (HR = 0.60, 95% CI = 0.51–0.71, *p* < 0.001) (Fig. 2d) (Supplementary Table 3).

### Major complications

A total of nineteen studies reported major complications (Supplementary Table 5A). Overall, the rates of major complications in the TACE combined with RFA group, the TACE group, and the RFA group were 2.71% (35/1291), 1.55% (20/1292), and 1.71% (12/701), respectively. The most observed major complications were gastrointestinal bleeding, abscess, liver failure, hepatic infarction, etc. (Supplementary Table 5B).

**Fig. 3:**
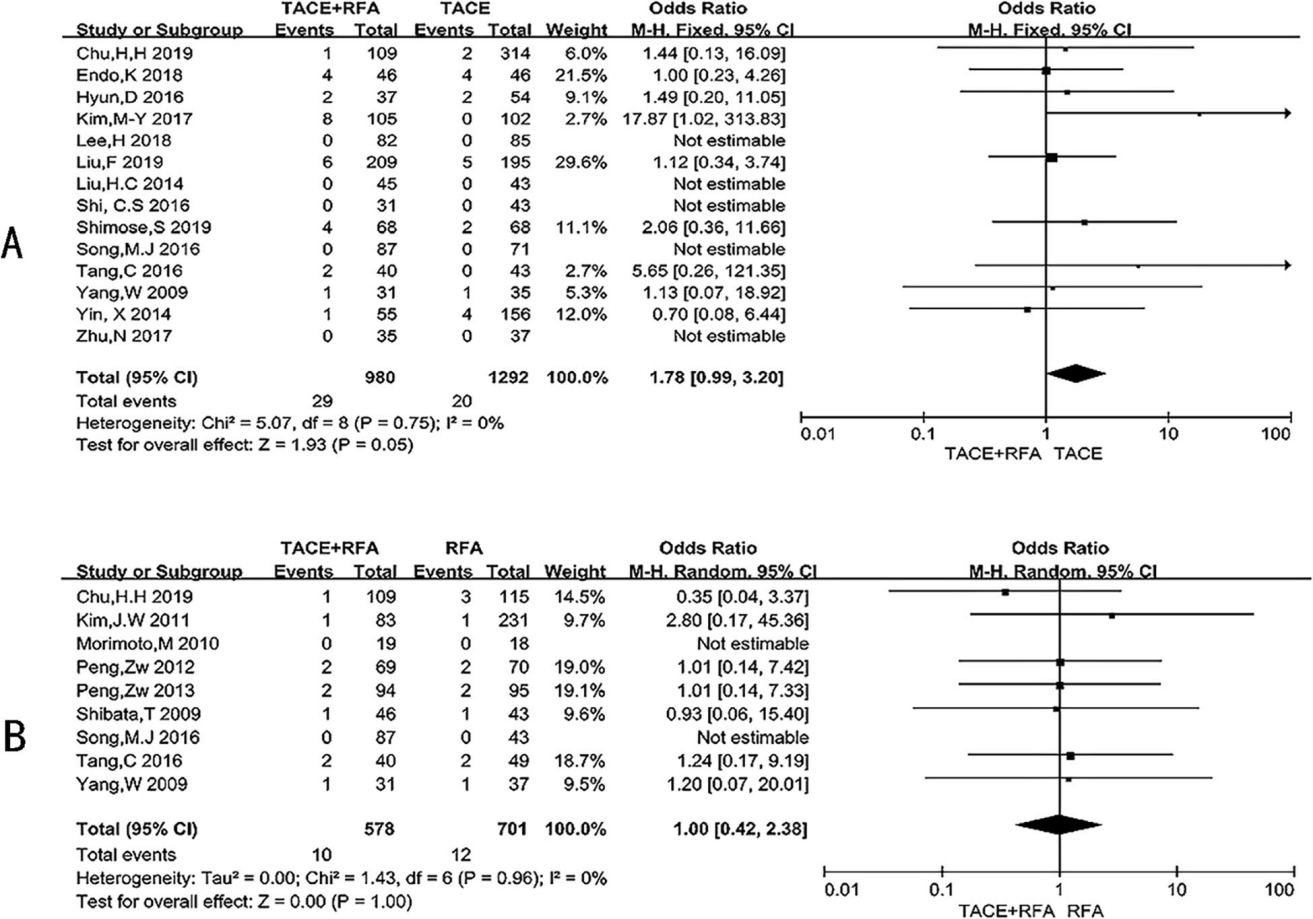
Meta-analysis of major complications. **a** TACE + RFA vs TACE; **b** TACE + RFA vs RFA

Fourteen studies with 2272 patients provided 49 major complications cases after TACE+RFA or TACE alone. The incidence of moderate to severe adverse effects for combined treatment was 2.96% (29/980), compared with only 1.55%(20/1292) in TACE monotherapy (OR = 1.78, 95% CI = 0.99–3.20, *p* = 0.05) (Fig. 3a) (Supplementary Table 4). Another group of nine studies, 1279 patients included, revealed similar rates of major complications of TACE+RFA vs RFA (1.73% (10/578) vs 1.71% (12/701)) (OR = 1.00, 95% CI = 0.42–2.38, *p* = 1.00) (Fig. 3b) (Supplementary Table 4) (Supplementary Table 5A).

### Subgroup analysis

#### Subgroup of tumors of diameter ≤3 cm

This subgroup results show that the combined treatment significantly improved OS and RFS (OS: HR=0.57, 95% CI=0.41–0.81, *p*=0.002; RFS: HR=0.47, 95% CI=0.30–0.73, *p*<0.001) compared with TACE alone. However, the combined group did not have better OS and RFS than RFA alone. (OS: HR=0.77, 95% CI=0.55–1.09, *p*=0.14; RFS: HR=0.82, 95% CI=0.57–1.19, *p*=0.30) alone (Table 2) (Supplementary Figure 3).

**Table 2:** Subgroup analysis based on the tumor size and the age

Criteria	OS					RFS				
	NO. of studies	NP	HR (95% CI)	*I*^2^	*p* value *Q* test	No. of studies	NP	HR (95% CI)	*I*^2^	*p* value *Q* test
TACE+RFA vs. TACE										
≤ 3cm	4	623	0.57 (0.41–0.81)	24%	0.27	2	365	0.47 (0.30–0.73)	62%	0.10
> 3cm	9	1238	0.61 (0.52–0.73)	0%	0.63	3	598	0.56 (0.35–0.89)	67%	0.05
Age < 60	6	783	0.61 (0.51–0.73)	0%	0.57	1	83	0.51 (0.30–0.87)	NA	NA
Age ≥ 60	6	834	0.58 (0.46–0.74)	0%	0.74	4	531	0.46 (0.36–0.57)	9%	0.35
TACE+RFA vs. RFA										
≤ 3cm	3	533	0.77(0.55–1.09)	22%	0.28	3	533	0.82 (0.57–1.19)	0%	0.91
> 3cm	6	669	0.61 (0.49–0.76)	0%	0.54	5	678	0.55 (0.45–0.67)	0%	0.47
Age < 60	5	799	0.61 (0.49–0.77)	41%	0.15	4	731	0.55 (0.43–0.70)	0%	0.68
Age ≥ 60	4	318	0.72 (0.49–1.04)	0%	0.51	4	318	0.80 (0.58–1.11)	0%	0.63

*TACE* transcatheter arterial chemoembolization, *RFA* radiofrequency ablation, *NP* number of patients, *NA* not applicable, *HR* hazard ratio, *OS* overall survival, *RFS* recurrence-free survival

#### Subgroup of tumors of diameter >3 cm

Meta-analysis of comparing TACE+RFA with TACE or RFA alone for tumors diameter larger than 3 cm showed that the combined group have better OS and RFS than TACE (OS: HR=0.61, 95% CI=0.52–0.73, *p*<0.001; RFS: HR=0.56, 95% CI= 0.35–0.89, *p*=0.01) or RFA (OS: HR=0.61, 95% CI=0.49–0.76, *p*<0.001; RFS: HR=0.55, 95% CI= 0.45–0.67, *p*<0.001) alone (Table 2) (Supplementary Figure 3).

#### Subgroup of age <60

Meta-analysis of comparing TACE+RFA with TACE or RFA alone for age <60 showed that the combined group have better OS and RFS than TACE (OS: HR=0.61, 95% CI=0.51–0.73, *p*<0.001; RFS: HR=0.51, 95% CI= 0.30–0.87, *p*=0.01) or RFA (OS: HR=0.61, 95% CI=0.49–0.77, *p*<0.001; RFS: HR=0.55, 95% CI= 0.43–0.70, *p*<0.001) alone (Table 2) (Supplementary Figure 4).

#### Subgroup of age ≥60

Meta-analysis of comparing TACE+RFA with TACE alone for age ≥60 shows that the combined treatment significantly improved OS and RFS (OS: HR=0.58, 95% CI=0.46–0.74, *p*<0.001; RFS: HR=0.46, 95% CI= 0.36–0.57, *p*<0.001) compared with TACE alone. However, the combined group has similar OS and RFS as RFA alone (OS: HR=0.72, 95% CI=0.49–1.04, *p*=0.08; RFS: HR=0.80, 95% CI= 0.58–1.11, *p*=0.19) alone (Table 2) (Supplementary Figure 4).

#### Publication bias and sensitivity analysis

**Fig. 4:**
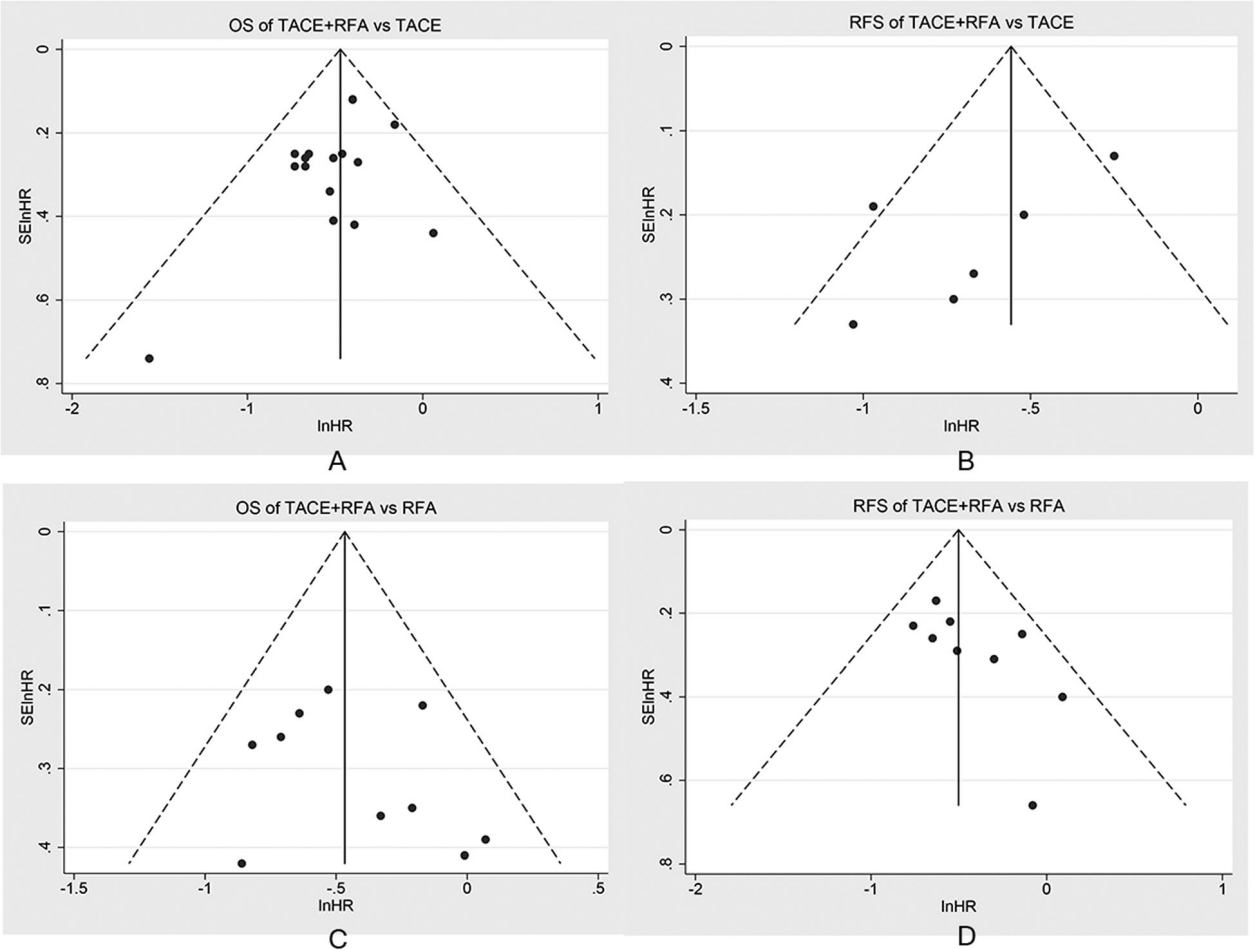
Publication bias evaluated by the funnel plots of TACE+RFA vs TACE or RFA alone for HCC. **a** OS of TACE+RFA vs TACE; **b** RFS of TACE+RFA vs TACE; **c** OS of TACE+RFA vs RFA; **d** RFS of TACE+RFA vs RFA

Funnel plots, Begg’s test, and Egger’s test were used to assess potential publication bias in this meta-analysis. The four funnel plots were roughly symmetrical on both sides (Fig. 4). In the group of TACE+RFA vs TACE, the studies yielded a Begg’s test score of *p*=0.727 and an Egger’s test score of *p*=0.143, and similar results were found for RFS (*p*=0.260 and 0.109, respectively); in the group of TACE+RFA vs RFA, the studies yielded a Begg’s test score of *p*=1.000 and an Egger’s test score of *p*=0.438, similar results were found for RFS (*p*=0.118 and 0.073, respectively), and so it can be considered that there was little publication bias in this meta-analysis.

**Fig. 5:**
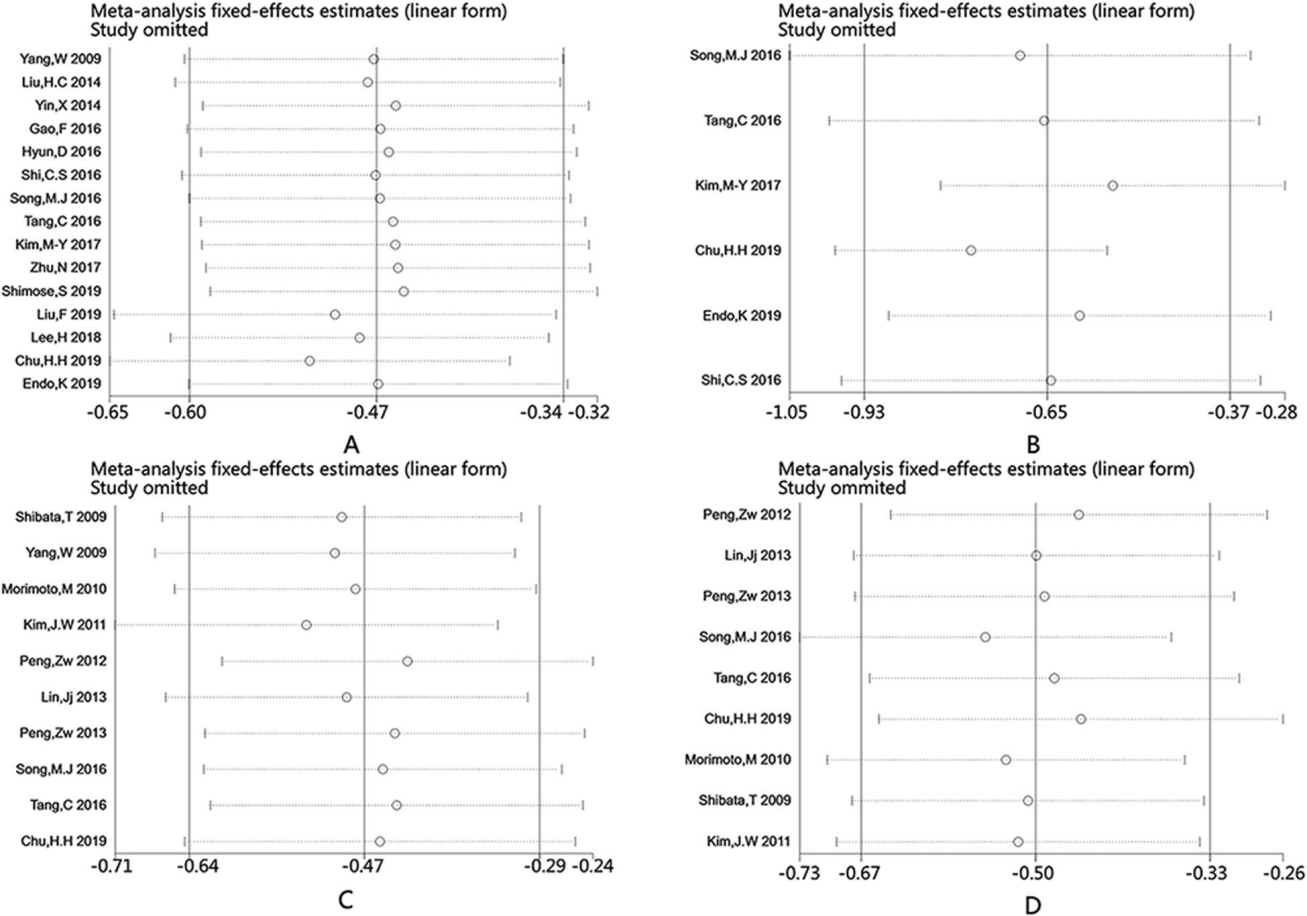
The sensitive analysis of TACE+RFA vs TACE or RFA alone for HCC. **a** OS of TACE+RFA vs TACE; **b** RFS of TACE+RFA vs TACE; **c** OS of TACE+RFA vs RFA; **d** RFS of TACE+RFA vs RFA. The *x*-axis refers to HR (hazard ratio)

Sensitivity analysis was performed by excluding individual studies to assess the impact of single studies on the stability of the combined results (Fig. 5), and we found that the combined results did not change after excluding any study. Therefore, the pooled results of this meta-analysis have been shown to be stable.

## Discussion

TACE and RFA are widely used in the treatment of HCC, but their application is controversial due to their corresponding deficiencies. In theory, the combination could improve treatment outcomes. TACE is usually recommended as the first choice for treatment of patients with BCLC Intermediate Stage (Stage B) disease that is inoperable. As a bridge treatment, some patients have the opportunity to undergo liver resection, RFA, or liver transplantation following TACE [55, 56]. Normal liver tissue has a double blood supply, both arterial and venous. However, HCC tissue is mainly supplied by arteries. In TACE, the chemotherapeutic drugs and embolization agents block the tumor blood supply, leading to tumor ischemia and hypoxia, which can inhibit tumor growth and promote tumor necrosis and apoptosis. However, hypoxia following embolization can stimulate the release of vascular endothelial growth factor (VEGF), which promotes the formation of new blood vessels in tumor areas [57–59]. Therefore, local recurrence is the main type after TACE treatment [60]. The RFA technique leads to coagulation necrosis in tumor tissue through heat, killing tumor cells. However, it is not only necessary to destroy tumor tissue, but also to ablate more than 1 cm of tissue at the edge of the tumor, so as to eliminate microsatellite foci and prevent recurrence [61]. Studies have shown that in cases of tumors larger than 3 cm with incomplete ablation, the risk of local recurrence is increased [62]. The heat-sink effect may be a reason for incomplete RFA ablation. The rich blood supply to the tumor tissue takes away the heat, thus reducing the therapeutic effect of RFA [63].

The use of TACE prior to RFA can block the blood supply of the tumor, minimize the heat loss caused by the heat-sink effect, increase the area of coagulation necrosis, produce more thorough internal necrosis of the mass, and expand the edge of the ablation to destroy the satellite lesions [15, 64, 65]. TACE is generally suitable for highly vascularized liver cancer, and this tumor property will increase the heat-sink effect of RFA therapy. Therefore, combined use has significant advantages [66]. In addition, as a regional treatment, TACE can identify satellite lesions missed by imaging, thus providing a target for subsequent RFA treatment [47]. At the same time, RFA treatment after TACE can kill tumor additional cells, destroy new blood vessels, and reduce tumor recurrence. Previous studies have shown that RFA can increase the deposition of some chemotherapeutic agents [67]. The research of Ako et al. shows that RFA treatment can achieve better clinical benefit at 7–20 days after TACE [68]. Such evidence shows that TACE combined with RFA in the treatment of HCC, especially in larger than 3 cm tumor, can reduce the recurrence rate and prolong the survival time.

So far, there have been some meta-analyses. Wang et al. [69] reported the meta-analysis comparing OS for patients receiving TACE+RFA and RFA alone in HCC. They analyzed six studies with 534 patients and showed similar OS and RFS. Yang et al. [21] investigated the outcome of TACE+RFA compared to TACE alone in HCC patients. They showed a survival benefit for combined therapy compared to TACE alone (OR_1-year_ = 3.92, 95% CI = 2.41–6.39, *p*<0.00001; OR_3-year_ = 2.56; 95% CI = 1.81–3.60; *p*<0.00001; OR_5-year_= 2.78, 95% CI= 1.77–4.38; *p* < 0.0001). However, previous meta-analyses only compared the efficacy of combination therapy versus either TACE or RFA, instead of making comparisons together. And a number of high-quality studies have been published in recent years; in addition, our study removed studies that were included in previous meta-analyses but were judged to be of low quality. As we know, our study is the largest meta-analysis including high-quality research through reasonable quality evaluation and subgroup analysis with clinical significance.

The result of this meta-analysis indicates that the OS and RFS rates of TACE combined with RFA are higher than those achieved with TACE alone (HR=0.62, 95% CI: 0.55–0.71, *p*<0.001; HR=0.52, 95% CI=0.39–0.69, *p*<0.001, respectively). The subgroup analysis results were the same as the overall results. The specific analysis also showed that although there was no significant difference of complications between the combined treatment group and the mono-therapy group, and the overall adverse events were prominent phenomena for patients who received associated therapies. This meta-analysis showed that TACE+RFA was better than RFA alone in the treatment of HCC in terms of both OS and RFS (HR=0.63, 95% CI: 0.53–0.75, *p*<0.001; HR=0.60, 95% CI=0.51–0.71, *p*<0.001, respectively), but in a subgroup of age ≥60 and tumors diameter ≤3 cm, there was no significant difference in OS and RFS between the combined group and the RFA alone group, so maybe RFA treatment is enough for these patients, but further research is needed to support this conclusion. In order to find the source of heterogeneity and eliminate it, we have performed a descriptive statistics analysis of clinicopathological characteristics of patients in 21 included studies (Supplementary Table 2) and found that some differences, such as country, tumor size, and Child-Pugh classification, which may be caused by different sources and inclusion criteria of various studies; these can be the sources of heterogeneity. Therefore, we selected the indicators that may have an impact on the prognosis for subgroup analysis. Then, we divided the studies into subgroups based on age and tumor size, and heterogeneity remained in subgroups based on tumor size, but decreased significantly in subgroups based on age; so, we hypothesized that heterogeneity might be due to the age of patients enrolled in different studies. Therefore, the results may need to be treated with caution.

Previous research reported that there was no significant difference in major complications between the combined treatment group and the RFA alone group [21]. The current meta-analysis shows a similar outcome (OR=1.00, 95% CI=0.42–2.38, *p*=1.00). Regarding major complications of TACE+RFA vs TACE, this meta-analysis also shows similar pooled outcomes (OR=1.78, 95% CI: 0.99–3.20, *p*=0.05) as another study [69]. Two patients died of hemorrhagic shock and liver failure, respectively, in the combined group and the TACE alone group [16, 36]. Although local treatment can result in a maximal reduction of systemic adverse reactions and trauma, fever, abdominal pain, fatigue, bone marrow depression, and other systemic manifestations are still common complications. Procedural safety is evaluated by the occurrence of serious complications, which is obviously not a comprehensive measure. Moreover, the definitions of severe complications are different in each study.

In a previously published meta-analysis, it was reported to be controversial to evaluate OS and RFS with ORs such as 1 year, 3 years, and 5 years [21]. Firstly, OR only describes information at a certain time point, so it cannot describe the entire process well. Next, the follow-up time was inconsistent in the original studies, which aggravated the occurrence of heterogeneity. In this study, HR was used to evaluate the time-to-event outcomes. In addition, some previous research did not use reasonable methods to evaluate the quality of literature [70]. In the present meta-analysis, the risk of bias was used to evaluate RCTs, and the NOS scale was used to evaluate cohort studies. As much as possible, research of higher quality was incorporated to evaluate TACE combined with RFA for the treatment of HCC. This meta-analysis use HR to assess the prognosis and safety of TACE+RFA and TACE or RFA alone in the treatment of HCC.

The present meta-analysis has some limitations. Firstly, although twenty-one studies were included, there were only three RCTs, so the quality of the evidence was relatively low. Secondly, all participants in the original studies were Asian, and eleven of these studies were conducted in China. Because HCC in China is often associated with hepatitis B (HBV), in contrast to the prevalence of HCV infection in Western countries, the results of this meta-analysis may not be applicable to other populations. Even in Asian countries, there was some heterogeneity in clinicopathological characteristics of patients such as Child-Pugh classification, tumor size, nationality, etc. due to different inclusion criteria and sources among studies. Owing to the lack of sufficient information in the original research, this meta-analysis was unable to include evaluation on the basis of different tumor stages. Lastly, owing to the small number of original studies in the subgroup analysis, the credibility of the results may be questionable. Therefore, additional high-quality RCTs and studies in other ethnic groups are needed to further explore TACE combined with RFA in the treatment of HCC.

## Conclusion

TACE combined with RFA might achieve better outcome for HCC patients compared with applied individually; moreover, the incidence of major complications with combined treatment was not increased compared to that with treatment alone, but we need further clinical trials to provide more evidence for this treatment attempt.

## Supplementary Information

**Additional file 1: Supplementary Figure 1**. The necessary steps of the methodology.

**Additional file 2: Supplementary Figure 2**. Risk of bias assessment of included RCTs.

**Additional file 3: Supplementary Figure 3**. The subgroup analysis of TACE+RFA vs TACE or RFA alone for HCC based on the tumor size. (A) TACE+RFA vs TACE:OS;(B)TACE+RFA vs TACE:RFS;(C)TACE+RFA vs RFA:OS;(D)TACE+RFA vs RFA:RFS.

**Additional file 4: Supplementary Figure 4**. The subgroup analysis of TACE+RFA vs TACE or RFA alone for HCC based on age. (A) TACE+RFA vs TACE:OS;(B)TACE+RFA vs TACE:RFS;(C)TACE+RFA vs RFA:OS;(D)TACE+RFA vs RFA: RFS.

**Additional file 5: Supplementary Table 1**. The NOS quality assessment of included cohort studies.

**Additional file 6: Supplementary Table 2**. Clinicopathological characteristics of patients in included studies.

**Additional file 7: Supplementary Table 3**. The OS and RFS of TACE+RFA vs TACE or RFA.

**Additional file 8: Supplementary Table 4**. The comparison of major complication of TACE+RFA vs TACE or RFA.

**Additional file 9: Supplementary Table 5A**. Major complications reported among the included studies.

**Additional file 10: Supplementary Table 5B**. Details of Major Complications among Included Studies.

## Data Availability

All the data analyzed in this study are obtained from the original articles.

## Abbreviations

TACE: Transcatheter arterial chemoembolization
RFA: Radiofrequency ablation
HCC: Hepatocellular carcinoma
CNKI: China National Knowledge Infrastructure
OS: Overall survival
RFS: Recurrence-free survival
HR: Hazard ratio
CI: Confidence intervals
RCTs: Randomized controlled trials
NOS: Newcastle Ottawa Quality Assessment Scale
OR: Odds ratio
VEGF: Vascular endothelial growth factor
