# Supplementary material for: Individual or combined transcatheter arterial chemoembolization and radiofrequency ablation for hepatocellular carcinoma: a time-to-event meta-analysis
Source: World J Surg Oncol. 2021 Mar 19;19:81. doi: 10.1186/s12957-021-02188-4 (PMC7980330; doi:10.1186/s12957-021-02188-4)
Supplement: Supplementary file 1 — Additional file 1: Supplementary Figure 1. The necessary steps of the methodology. [file 12957_2021_2188_MOESM1_ESM.docx]

Supplementary Table 1:The NOS quality assessment of cohort studies

| Study | Type | Study arms | Representativeness of exposed cohort | Selection of the non-exposed cohort | Ascertainment of exposure | Demonstration that outcome of interest was not present at the start | Comparability of cohorts on the basis of the design | Assessment of outcome | Was follow-up long enough for outcomes to occur | Adequacy of follow-up of cohort | Total |
| --- | --- | --- | --- | --- | --- | --- | --- | --- | --- | --- | --- |
| Shibata, T.2009 | cohort study | TACE+RFA,RFA | * | * | * | * | ** | * | * |  | 8 |
| Yang, W 2009 | cohort study | TACE+RFA,TACE,RFA | * | * | * | * | ** | * | * |  | 8 |
| Kim, J. W 2011 | cohort study | TACE+RFA , RFA | * | * | * | * | ** | * | * |  | 8 |
| Lin, Jj2013 | cohort study | TACE+RFA ,RFA | * | * | * | * | * | * | * |  | 7 |
| Liu, H. C 2014 | cohort study | TACE+RFA,TACE | * | * | * | * | * | * | * |  | 7 |
| Yin, X 2014 | cohort study | TACE+RFA,TACE | * | * | * | * | ** | * | * |  | 8 |
| Gao, F 2016 | cohort study | TACE+RFA,TACE | * | * | * | * | ** | * | * |  | 8 |
| Hyun, D 2016 | cohort study | TACE+RFA,TACE | * | * | * | * | ** | * | * |  | 8 |
| Shi, C. S 2016 | cohort study | TACE+RFA,TACE | * | * | * | * | ** | * | * |  | 8 |
| Song, M. J 2016 | cohort study | TACE+RFA,TACE,RFA | * | * | * | * | ** | * | * |  | 8 |
| Tang, C 2016 | cohort study | TACE+RFA,TACE,RFA | * | * | * | * | ** | * | * |  | 8 |
| Kim, M-Y 2017 | cohort study | TACE+RFA,TACE | * | * | * | * | ** | * | * | * | 9 |
| Zhu, N 2017 | cohort study | TACE+RFA,TACE | * | * | * | * | ** | * | * |  | 8 |
| Shimose,S 2019 | cohort study | TACE+RFA,TACE | * | * | * | * | ** | * | * |  | 8 |
| Liu, F 2019 | cohort study | TACE+RFA,TACE | * | * | * | * | ** | * | * |  | 8 |
| Lee,H 2018 | cohort study | TACE+RFA,TACE | * | * | * | * | ** | * | * |  | 8 |
| Chu,H,H 2019 | cohort study | TACE+RFA,TACE,RFA | * | * | * | * | ** | * | * |  | 8 |
| Endo,K 2018 | cohort study | TACE+RFA,TACE | * | * | * | * | ** | * | * |  | 8 |

TACE:transcatheter arterial chemoembolization,RFA:radiofrequency ablation,NOS :Newcastle-Ottawa scale
