# Supplementary material for: Individual or combined transcatheter arterial chemoembolization and radiofrequency ablation for hepatocellular carcinoma: a time-to-event meta-analysis
Source: World J Surg Oncol. 2021 Mar 19;19:81. doi: 10.1186/s12957-021-02188-4 (PMC7980330; doi:10.1186/s12957-021-02188-4)
Supplement: Supplementary file 2 — Additional file 2: Supplementary Figure 2. Risk of bias assessment of included RCTs. [file 12957_2021_2188_MOESM2_ESM.docx]

Supplementary Table 2:Clinicopathological characteristics of patients in included studies.

|  | TACE+RFA vs TACE(n=2339) | | | | | TACE+RFA vs RFA(n=1341) | | | |
| --- | --- | --- | --- | --- | --- | --- | --- | --- | --- |
|  | TACE+RFA  （n=1015） | TACE  （n=1324） | | *P* value | | TACE+RFA  （n=610） | RFA  （n=731） | *P* value | |
| gender（M/F） | 788/227 | 1008/316 | | 0.401 | | 480/130 | 558/173 | 0.326 | |
| age（years） | 59.91±10.7 | 59.96±11.25 | | 0.919 | | 58.54±11.2 | 58.48±11.87 | 1.000 | |
| country |  |  | | 0.007 | |  |  | 0.02 | |
| Japan | 114 | 114 | |  |  | 65 | 61 |  |  |
| Korea | 420 | 626 |  | |  | 279 | 389 |  |  |
| China | 481 | 584 | |  |  | 266 | 281 |  |  |
| tumor Size(cm) | 3.23±1.24 | 3.07±1.26 | | 0.03 | | 3.66±1.64 | 4.03±1.66 | <0.001 | |
| HBsAg+ | 564 | 795 | | 0.097 | | 363 | 443 | 0.903 | |
| HCV-Ab+ | 174 | 173 | | 0.019 | | 113 | 109 | 0.04 | |
| Child-Pugh |  |  | | 0.188 |  |  |  | 0.013 |  |
| A | 783 | 1014 | |  | | 478 | 533 |  | |
| B | 151 | 231 | |  |  | 99 | 167 |  |  |
| C | 13 | 11 | |  |  | 1 | 1 |  |  |

TACE:transcatheter arterial chemoembolization,RFA:radiofrequency ablation,M/F:male/famale
