# Supplementary material for: Individual or combined transcatheter arterial chemoembolization and radiofrequency ablation for hepatocellular carcinoma: a time-to-event meta-analysis
Source: World J Surg Oncol. 2021 Mar 19;19:81. doi: 10.1186/s12957-021-02188-4 (PMC7980330; doi:10.1186/s12957-021-02188-4)
Supplement: Supplementary file 3 — Additional file 3: Supplementary Figure 3. The subgroup analysis of TACE+RFA vs TACE or RFA alone for HCC based on the tumor size. (A) TACE+RFA vs TACE:OS;(B)TACE+RFA vs TACE:RFS;(C)TACE+RFA vs RFA:OS;(D)TACE+RFA vs RFA:RFS. [file 12957_2021_2188_MOESM3_ESM.docx]

Supplementary Table 3:The OS and RFS of TACE+RFA vs TACE or RFA.

| Groups | OS | | | | RFS | | | |
| --- | --- | --- | --- | --- | --- | --- | --- | --- |
|  | NO.of studies | NP | HR(95% CI) | P value | NO. of studies | NP | HR(95% CI) | P value |
| TACE+RFA vs TACE | 15 | 2339 | 0.62(0.55-0.71) | <0.001 | 6 | 1037 | 0.52(0.39-0.69) | <0.001 |
| TACE+RFA vs RFA | 10 | 1341 | 0.63(0.53-0.75) | <0.001 | 9 | 1273 | 0.60(0.51-0.71) | <0.001 |

NP:Number of Patients, NA: not applicable, HR:hazard ratio,95% CI:95% confidence intervals,OS:overall survival,RFS:recurrence-free survival
