# Supplementary material for: Individual or combined transcatheter arterial chemoembolization and radiofrequency ablation for hepatocellular carcinoma: a time-to-event meta-analysis
Source: World J Surg Oncol. 2021 Mar 19;19:81. doi: 10.1186/s12957-021-02188-4 (PMC7980330; doi:10.1186/s12957-021-02188-4)
Supplement: Supplementary file 4 — Additional file 4: Supplementary Figure 4. The subgroup analysis of TACE+RFA vs TACE or RFA alone for HCC based on age. (A) TACE+RFA vs TACE:OS;(B)TACE+RFA vs TACE:RFS;(C)TACE+RFA vs RFA:OS;(D)TACE+RFA vs RFA: RFS. [file 12957_2021_2188_MOESM4_ESM.docx]

| Groups | Major complications | | | | |
| --- | --- | --- | --- | --- | --- |
|  | NO.of studies | NP | Incidence  comparison | OR(95% CI) | P value |
| TACE+RFA vs TACE | 14 | 2272 | 2.96% vs 1.55% | 1.78(0.99-3.20) | 0.05 |
| TACE+RFA vs RFA | 9 | 1279 | 1.73% vs 1.71% | 1.00(0.42-2.38) | 1.00 |

Supplementary Table 4:The comparison of major complication of TACE+RFA vs TACE or RFA.

NP:Number of Patients, NA: not applicable, HR:hazard ratio,95% CI:95% confidence intervals
