# Supplementary material for: Individual or combined transcatheter arterial chemoembolization and radiofrequency ablation for hepatocellular carcinoma: a time-to-event meta-analysis
Source: World J Surg Oncol. 2021 Mar 19;19:81. doi: 10.1186/s12957-021-02188-4 (PMC7980330; doi:10.1186/s12957-021-02188-4)
Supplement: Supplementary file 5 — Additional file 5: Supplementary Table 1. The NOS quality assessment of included cohort studies. [file 12957_2021_2188_MOESM5_ESM.docx]

Supplementary Table 5A:Major Complications reported among the Included Studies

| Study | Major complications | | |
| --- | --- | --- | --- |
|  | TACE+RFA | TACE | RFA |
| Shibata,T 2009 | 1/46 | NA | 1/43 |
| Yang,W 2009 | 1/31 | 1/35 | 1/37 |
| Morimoto,M 2010 | 0/19 | NA | 0/18 |
| Kim,J. W 2011 | 1/83 | NA | 1/231 |
| Peng,Zw 2012 | 2/69 | NA | 2/70 |
| Peng,Zw 2013 | 2/94 | NA | 2/95 |
| Liu,H.C 2014 | 0/45 | 0/43 | NA |
| Yin,X 2014 | 1/55 | 4/156 | NA |
| Hyun,D 2016 | 2/37 | 2/54 | NA |
| Shi,C.S 2016 | 0/31 | 0/43 | NA |
| Song, M. J 2016 | 0/87 | 0/71 | 0/43 |
| Tang,C 2016 | 2/40 | 0/43 | 2/49 |
| Kim,M-Y 2017 | 8/105 | 0/102 | NA |
| Zhu,N 2017 | 0/35 | 0/37 | NA |
| Shimose,S 2019 | 4/68 | 2/68 | NA |
| Liu, F 2019 | 6/209 | 5/195 | NA |
| Lee,H 2018 | 0/82 | 0/85 | NA |
| Chu,H.H 2019 | 1/109 | 2/314 | 3/115 |
| Endo,K 2018 | 4/46 | 4/46 | NA |

TACE:transcatheter arterial chemoembolization,RFA:radiofrequency ablation,NA:not applicable
