# Supplementary material for: Individual or combined transcatheter arterial chemoembolization and radiofrequency ablation for hepatocellular carcinoma: a time-to-event meta-analysis
Source: World J Surg Oncol. 2021 Mar 19;19:81. doi: 10.1186/s12957-021-02188-4 (PMC7980330; doi:10.1186/s12957-021-02188-4)
Supplement: Supplementary file 6 — Additional file 6: Supplementary Table 2. Clinicopathological characteristics of patients in included studies. [file 12957_2021_2188_MOESM6_ESM.docx]

Supplementary Table 5B:Details of Major Complications among Included Studies

| Study | Major complications | | |
| --- | --- | --- | --- |
|  | TACE+RFA | TACE | RFA |
| Shibata,T 2009 | segmental hepatic infarction(n=1) | NA | subcapsular hemorrhage of pseudoaneurysm(n=1) |
| Yang,W 2009 | Hemothorax(n=1) | liver function failure (n=1)(die) | Moderate intraperitoneal hemorrhage(n=1) |
| Morimoto,M 2010 | 0 | NA | 0 |
| Kim,J. W 2011 | Bleeding and hypovolemic shock(n=1)(die) | NA | biloma(n=1) |
| Peng,Zw 2012 | moderate ascites(n=1), liver failure(n=1) | NA | severe ascites(n=1),persistent jaundice(n=1) |
| Peng,Zw 2013 | Bile duct stenosis(n=1),Gastric hemorrhage (n=1) | NA | Abdominal infection(n=1),Small intestinal obstruction(n=1) |
| Liu,H.C 2014 | 0 | 0 | NA |
| Yin,X 2014 | Gastrointestinal bleeding(n=1) | liver dysfunction(n=2),upper gastrointestinal bleeding(n=2) | NA |
| Hyun,D 2016 | biliary strictures(n=2) | Acute cholecystitis(n=1),hepatic failure(n=1) | NA |
| Shi,C.S 2016 | 0 | 0 | NA |
| Song, M. J 2016 | 0 | 0 | 0 |
| Tang,C 2016 | intraperitoneal hemorrhage(n=1), hemothorax(n=1) | 0 | intraperitoneal hemorrhage(n=2) |
| Kim,M-Y 2017 | Abscess(n=2);hemoperitoneum(n=2),  hepatic failure(n=1),delayed colon perforation(n=1),Pneumothorax (n=1),Segmental hepatic infarction (n=1) | 0 | NA |
| Zhu,N 2017 | 0 | 0 | NA |
| Shimose,S 2019 | hepatic hemorrhage (n=2), bile duct injury (n=2) | hepatic failure（n=2) | NA |
| Liu, F 2019 | Bile duct stenosis(n=2),Gastric hemorrhage(n=2),Abdominal infection(n=1),Small intestinal obstruction(n=1) | Bile duct stenosis(n=2),Gastric hemorrhage(n=2),Abdominal infection(n=1) | NA |
| Lee,H 2018 | 0 | 0 | NA |
| Chu,H.H 2019 | intraperitoneal bleeding(n=1) | HCC rupture(n=1),hepatic abscess(n=1) | cloinic microperforation(n=1),intraperitoneal bleeding(n=1),segmental hepatic infarction(n=1) |
| Endo,K 2018 | ileus (n=1), sub capsular hemorrhage of liver (n=1), cholecystitis (n=1) and pleural effusion (n=1) | pancreatitis (n=1), cholecystitis (n=1), and ascites (n=1) and pleural effusion (n=1). | NA |

TACE:transcatheter arterial chemoembolization,RFA:radiofrequency ablation,NA:not applicable
