# Supplementary figures and images for: Individual or combined transcatheter arterial chemoembolization and radiofrequency ablation for hepatocellular carcinoma: a time-to-event meta-analysis
Source: World J Surg Oncol. 2021 Mar 19;19:81. doi: 10.1186/s12957-021-02188-4 (PMC7980330; doi:10.1186/s12957-021-02188-4)

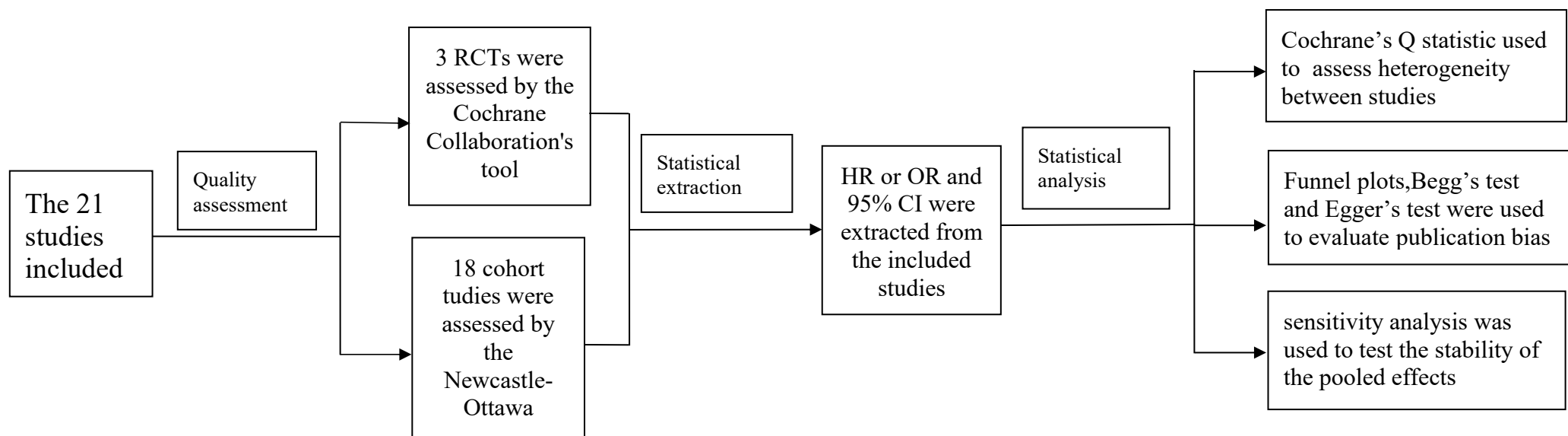

Supplement: Supplementary file 7 — Additional file 7: Supplementary Table 3. The OS and RFS of TACE+RFA vs TACE or RFA. [file 12957_2021_2188_MOESM7_ESM.pdf]

A

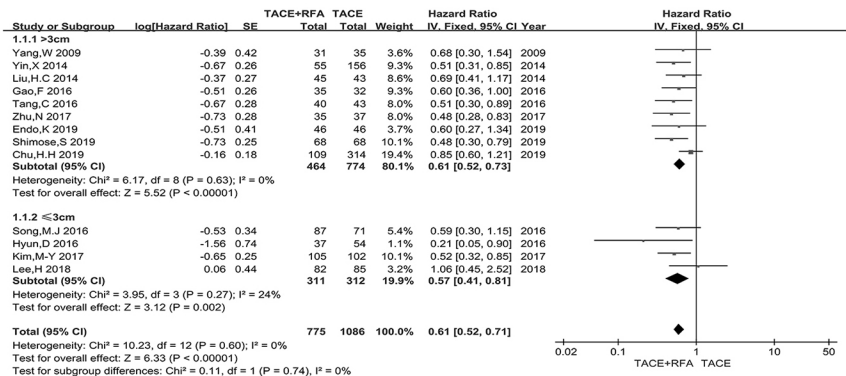

B

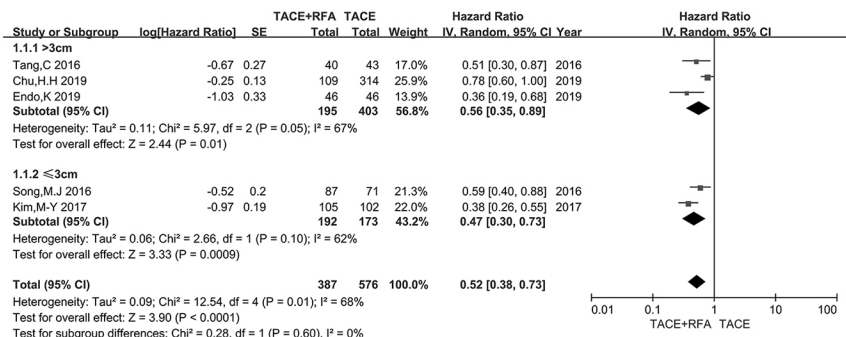

C

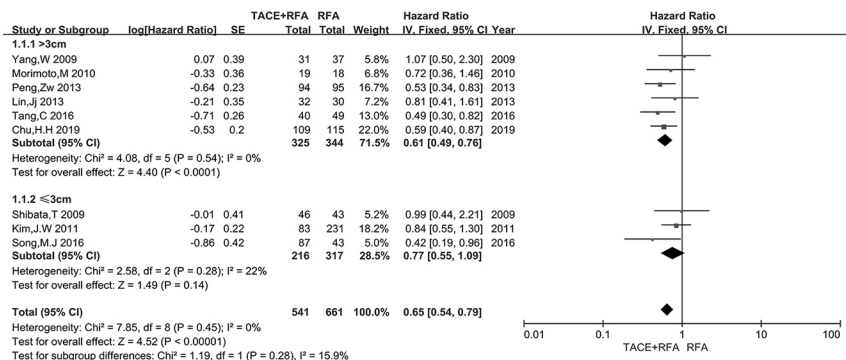

D

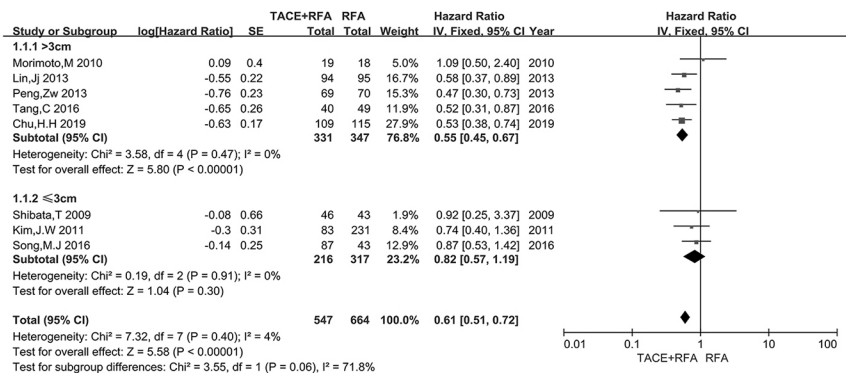

Supplement: Supplementary file 9 — Additional file 9: Supplementary Table 5A. Major complications reported among the included studies. [file 12957_2021_2188_MOESM9_ESM.pdf]

A

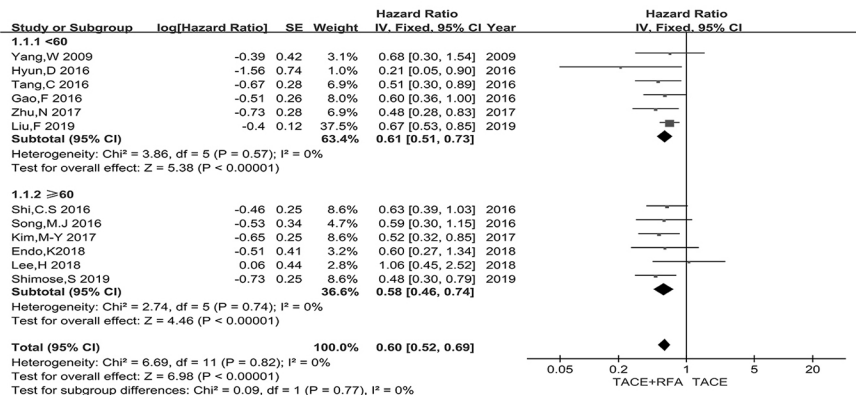

B

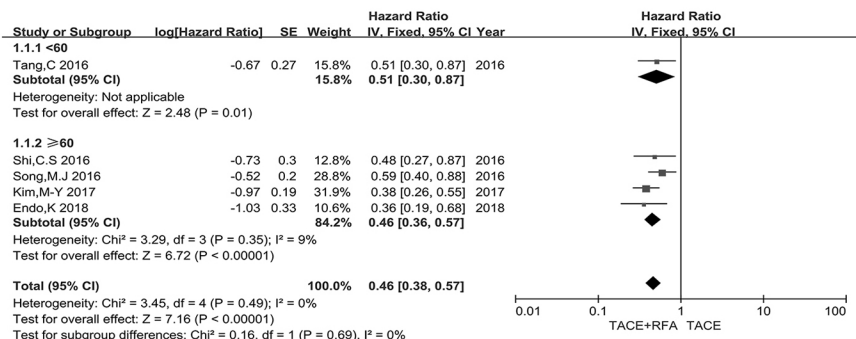

C

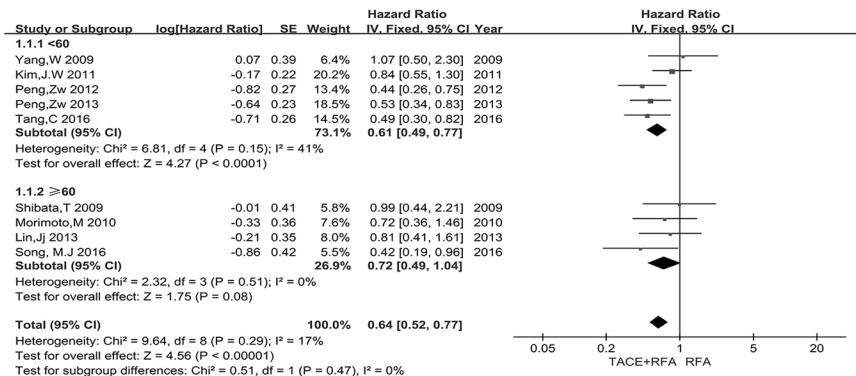

D

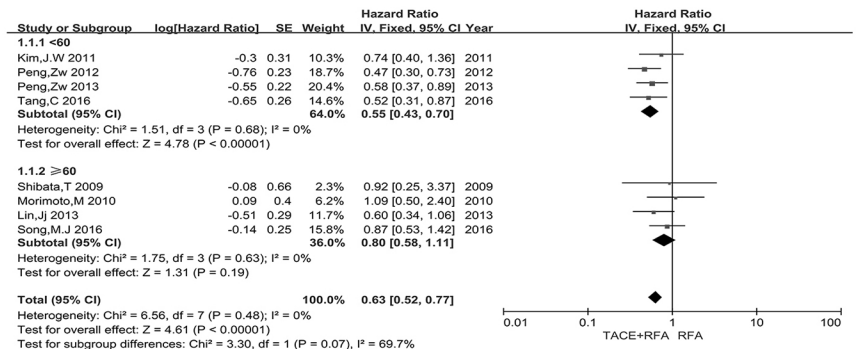

Supplement: Supplementary file 10 — Additional file 10: Supplementary Table 5B. Details of Major Complications among Included Studies. [file 12957_2021_2188_MOESM10_ESM.pdf]
